# 515 Prevotella Melaninogenica-derived Metabolites Induce Immune Reprogramming of Human THP-1 Macrophages

**DOI:** 10.1093/jbcr/iraf019.144

**Published:** 2025-04-01

**Authors:** Angel Charles, Denise Hernandez, Madelyn Bucci, Shannon Wallet, Robert Maile

**Affiliations:** University of Florida; University of Florida; University of Florida; University of Florida; University of Florida

## Abstract

**Introduction:**

Inhalation injury is responsible for ~90% of burn-related deaths. This is primarily due to innate immune dysfunction in the lungs, which leads to the development of acute lung injury (ALI) and pneumonia. Prevotella melaninogenica (Pm), has been shown to associate with the development of ALI after inhalation injury. We hypothesized that metabolites secreted by Pm can modulate expression of immune-related genes in monocytes.

**Methods:**

Pm was grown under anaerobic conditions in chopped meat medium for 48 hours. Human monocytic THP-1 cells were stimulated for 24 hours with Pm-derived cell-free culture supernatant (CFS). We also added 10ng/ml of LPS to some cultures to test the ability of Pm-CFS to re-program strong anti-microbial immune responses such as those during sepsis. RNA was isolated and we performed nanoString analysis to probe for multiple transcriptionally active genes (Human Immunology CodeSet, 594 mRNA immune genes. Data were analyzed by ROSALIND, with fold changes and p-values are calculated, with p-value adjustment based on estimating false discovery rates (FDR). We then utilized Ingenuity Pathway Analysis (IPA) to generate z-scores which represent the activation or inhibition state of a canonical pathway.

**Results:**

Unstimulated THP-1 cells treated with Pm-derived CFS exhibited a significant increase in the expression of 14 genes, and a significant decrease in the expression of 3 genes when compared with media control (p < 0.05). CXCL10 expression was increased x9.9 fold, CCL2 (MCP-1) increased x8.7 fold, and ICAM1 increased x6.4 fold. IPA indicated significant (p< 0.05) reprogramming of immune pathways, with significant increase in IL-10 Signaling (Z-score, +3.76). Pm-CFS also altered the immune transcriptional response to LPS, resulting in a significant increase in the expression of 29 genes and a significant decrease in the expression of 2 genes when compared to LPS alone (p < 0.05). TLR9 expression was increased 7.4 fold, and IL2RB expression increased 6.7 fold, with a corresponding increase in Pathogen Induced Cytokine Storm Signaling Pathway (z-score, +4.5).

**Conclusions:**

Pm products induced changes in immune-related gene expression in THP-1 cells. Studies are ongoing to determine the bacterial products present in Pm-CFS responsible for these phenotypes, and whether these immune changes translate into functional differences such as anti-bacterial responses or tissue damage associated with ALI.

**Applicability of Research to Practice:**

This study highlights the role of microbial-derived products in regulating monocyte function, as a possible therapeutic target to regulate the innate immune response in the airway after inhalation injury.

**Funding for the Study:**

T32 Interdisciplinary Training for Vascular Surgeon Scientists (ITVSS) 5T32HL160491-02

